# Cardiopulmonary Exercise Testing

**DOI:** 10.1155/2012/564134

**Published:** 2012-12-26

**Authors:** Luke Howard, Michael P. W. Grocott, Robert Naeije, Ron Oudiz, Roland Wensel

**Affiliations:** ^1^Imperial College School of Medicine, Imperial College London, London W12 0NN, UK; ^2^Centre for Human Integrative Physiology, University of Southampton, Southampton, UK; ^3^Erasme Academic Hospital, Free University of Brussels, Brussels, Belgium; ^4^Los Angeles Biomedical Research Institute at Harbor, UCLA Medical Center, Torrance, CA, USA

Cardiopulmonary exercise testing (CPET) is important for the differential diagnosis of dyspnoea-fatigue syndromes. The test more typically includes measurements of ventilation (*V*_*E*_), carbon dioxide output (VCO_2_), and oxygen uptake (VO_2_) at a progressively increased workload (*W*) until a maximum VO_2_, called VO_2max_ or VO_2peak_ to define aerobic exercise capacity, but steady state evaluations have utility in some contexts. The difference in VO_2max_ and VO_2peak_ can sometimes be made by the identification or not of a VO_2_ plateau while workload may be still increasing. However, the information content of VO_2max_ and VO_2peak_ is essentially the same, provided other criteria of maximum exercise are met, with, importantly, a respiratory exchange ratio (RER) ≥1.1. Ventilatory reserve may be calculated at VO_2peak_. This is the difference between maximum voluntary ventilation (MVV) and V_Epeak_, with MVV either directly measured, or predicted from the forced expiratory volume in 1 sec (FEV_1_) times 35 or 40. The ventilatory reserve normally ranges from 20 to 60 L/min, with an extreme lower limit of normal of 11 L/min, although caution should be applied to this measure given the unreliability of calculated MVV. Other relevant CPET measurements are VO_2_ at the anaerobic threshold (VO_2_AT) (RER = 1), *V*_*E*_/VCO_2_ either as a slope over the entire CPET or, preferably, at the AT, maximum heart rate and recovery, and the VO_2_-work rate relationship (ΔVO_2_/Δ*W*). Indeed *V*_*E*_/VCO_2_ slope has been shown to be a powerful prognostic indicator in heart failure independent of peak VO_2_.

Cardiac limitation of aerobic exercise capacity is characterized by decreased VO_2_AT, ΔVO_2_/Δ*W* and increased *V*_*E*_/VCO_2_-slope, when heart failure is present. In these patients, resting heart rate is increased, but maximum heart rate and heart rate recovery are decreased (the latter to <12 beats per min). A ventilatory limitation to exercise capacity is characterized by a low ventilatory reserve, which is often, but not always, attained with RER < 1. The CPET profile of severe deconditioning resembles that of heart failure, but with VO_2max_ usually >20 mL/kg except in the elderly and an unremarkable *V*_*E*_/VCO_2_. The identification of “peripheral factors” is more difficult. Mitochondrial disorders are uncommon causes of early lactic acidosis with very low VO_2max_ and VO_2_AT—along with a hyperdynamic cardiovascular state. Muscle mass in patients with chronic disease-associated cachexia may become insufficient for a maximal challenge of the cardiovascular system, which is normally achieved with approximately half of the skeletal muscle mass (legs on the bicycle). Peripheral oxygen extraction in heart failure has been shown to be preserved. Therefore, in these patients VO_2max_ is essentially maximal cardiac output (*Q*-) dependent, as indicated by the Fick equation:

(1) $$\begin{array}{c} \text{V}{\text{O}}_{2\max}=Q_{\max}\left(\text{Ca}{\text{O}}_{2}-\text{Cv}{\text{O}}_{2}\right), \end{array}$$

where CvO_2_ becomes a constant, and thus VO_2max_ determined by maximum O_2_ delivery or *Q*  ×  CaO_2_.

Many studies have reported on marked histological and biological changes in skeletal muscle of patients with chronic diseases, including heart failure, but measurements of maximum O_2_ extraction are few. Skeletal muscle deterioration in cardiac or lung disease patients may be most relevant to muscle strength or power, which is more appropriately measured by anaerobic exercise capacity tests.

A simple, inexpensive, and safe surrogate of VO_2max_ is the distance walked in 6 minutes. The 6-min walk test (6MWT) derives from the linear relationships between VO_2_, *Q* or workload,—or speed. Like VO_2max_, the 6-min walk distance is also decreased by decreases in CaO_2_ as a cause of decreased oxygen delivery and breathing reserve. The test is limited in the less severely impaired patients by a ceiling effect, corresponding to the maximum possible speed of walking in patients still be able to further increase VO_2_—but for that purpose would have to switch to walk on a slope or running. Demographic factors such as age, height and weight should also be taken in to consideration of the distance walked. The 6MWT is very useful to evaluate the functional impairment of patients, less so for the differential diagnosis of dyspnoea-fatigue. 

In the present issue of Pulmonary Medicine, these basic exercise physiology principles are further explored by nice series of clinical studies on patients with a variety of cardiac and pulmonary diseases.

In the study entitled “*Abnormalities of the ventilatory equivalent for carbon dioxide in patients with chronic heart failure*” L. Ingle et al. investigated the kinetics of the *V*_*E*_/VCO_2_ ratio during an incremental exercise testing following a modified Bruce protocol in a large series of 423 patients with chronic heart failure compared to 78 healthy controls. They showed that the nadir of the *V*_*E*_/VCO_2_ in patients with heart failure occurred earlier, and that this was of prognostic relevance. These results add to previously known prognostic value of increased *V*_*E*_/VCO_2_-slope in heart failure. The authors' interpretation of the findings is that a shorter time to *V*_*E*_/VCO_2nadir_ reflects earlier onset of the non-CO_2_ stimulus to ventilation in patients with chronic heart failure. This is possible. However, the time to *V*_*E*_/VCO_2nadir_ was decreased in proportion to the decreased exercise duration, VO_2_AT or VO_2peak_, all at around 60% of measured in the controls, indicating an important intrinsic contribution of aerobic exercise capacity, known as another determinant of survival in heart failure.

The study by R. L. Chura et al. “*Test-retest reliability and physiological responses associated with the steep ramp anaerobic test in patients with COPD*” provides robust data on the reproducibility of a very steep incremental exercise protocol (25 W per 10 s) in 11 COPD patients. The Steep Ramp Anaerobic test (SRAT) aimes at a measurement of anaerobic exercise capacity, and, as such, was compared by the authors to the more classically known 30s-Wingate Anaerobic Test (WAT). The patients also performed a CPET. Both SRAT and WAT showed a good reproducibility at 1-2 days intervals. Maximum power output was 157 W on the SRAT, compared to 231 W on the WAT and 66 W on the CPET, but metabolic and ventilatory responses were similar. The FEV_1_ of the patients was on average of 1 L, allowing to estimate a MVV of 40 L/min. The *V*_*E*max_ was on average 40 L/min during the three exercise tests, indicating that the three tests exhaused the ventilatory reserve of the patients. Because the peak work output was the highest at the maximum achieved VO_2_ with the SRAT, the authors concluded about the superiority of this test over the WAT to determine anaerobic exercise capacity. This makes a lot of sense. Furthermore, the higher power output with the SRAT compared to the WAT at similar VO_2_ probably reveals more specific measurement of purely alactic anaerobic exercise capacity. This important study will hopefully draw attention to the importance of anaerobic exercise testing in the evaluation of patients with cardiac and pulmonary diseases. 

In the article “*Cardiopulmonary exercise testing in lung transplantation: a review*” K. A. Dudley and S. El-Chemaly review published data on the role of CPET in the assessment of appropriateness of listing for lung transplantation as well of posttransplant outcome in patients with various forms of lung disease. The lung allocation score does not currently include CPET variables, but exercise capacity solely determined by a 6MWT. The review confirmed the previously known prognostic relevance of the 6MWT for patients awaiting transplantation. In these patients, the 6MWT was superior to spirometry in the prediction of 6-month mortality. However, how pretransplant 6MWT relates to posttransplant functional state and prognosis remains unclear. The reason why CPET variables are currently not components of the lung allocation score is in the insufficient evidence derived from limited size studies evaluating only specific respiratory pathologies and type of transplant, thus with findings that are difficult to extrtapolate to other patients waiting for transplantation. Data from posttransplant patients reveal some functional improvement with, however, still significant reduction in exercise tolerance. This does not appear to be limited by the pulmonary function, therefore pointing towards peripheral mechanisms of reduced functional capacity. Despite the need for more systematic studies in this field, the authors conclude that, based on the existing data, CPET carries prognostic information beyond that of pulmonary function tests and 6-minute-walk distance suggesting a role of CPET in pretransplant and possibly also posttransplant assessment. 

The study “*Predicted aerobic exercise capacity of asthmatic children: a research study from clinical origin*” by L. Lochte compared longitudinally the predicted aerobic exercise capacity based on submaximal exercise testing in 28 asthmatic children compared to 28 controls during 10 months. There were also physical activity and asthma questionnaires. The predicted aerobic exercise capacity test consisted in 5 min continuous running on a tredmill at a running speed adjusted to maintain a stable submaximal heart rate of 170–180 bpm. The estimation of VO_2max_ was based on previously reported weight-adjusted VO_2_, running speed and heart rate, and VO_2_ extrapolated from the difference between maximum and submaximum heart rates. This is an adaptation of the Astrand test, based on the same rationale that any further increase in VO_2_ (or *Q*) at a submaximum level of exercise is exclusively heart rate determined. Physical activity as evaluated by questionnaires was well correlated to estimated VO_2max_—and age. The results showed lower predicted aerobic exercise capacity in asthmatic children, without this being related to asthma severity or exercise induced asthma, but there was improvement over time. The reasons for decreased fitness in young asthmatics are unclear, but it might be deconditioning rather than insufficient asthma control. The study underscores the importance of monitoring physical activity in addition to lung function in children with asthma.

The study “*The impact of pulmonary arterial pressure on exercise capacity in mild-to-moderate cystic fibrosis: a case control study*” by K. Manika et al. explored the impact of systolic pulmonary artery pressure (PASP) measured by echocardiography before and immediately after testing on exercise capacity in 17 patients with mild-to-moderate cystic fibrosis without pulmonary hypertension and in 10 controls. The patients hads higher postexercise PASP and lower VO_2peak_, VO_2_AT, and peak O_2_ pulse than the controls. The change in PASP was strongly correlated to parameters of exercise capacity in the patients, not in the controls. The authors conclude that pulmonary vascular disease might contribute to decreased exercise capacity in patients with cystic fibrosis. It may be noted that the postexercise PASP were not higher than normal, and the difference between patients and controls, though significant, was of a few mmHg, thus in the range of the error of measurement. However, the patients had an average ventilatory reserve of around 40 L/min at maximum exercise, the maximum RER was of 1.19, not different from the controls and the patients with the highest PASP also had a higher *V*_*E*_/VCO_2_. It is thus possible that pulmonary vascular disease contributes to the limitation of exercise capacity in patients with cystic fibrosis. Further studies on a larger number of patients with more comprehensive measurements of the pulmonary circulation will be needed to prove it.

The study “*Validity of reporting oxygen uptake efficiency slope from submaximal exercise using respiratory exchange ratio as secondary criterion*” by W. Williamson et al. investigated the VO_2_ efficiency slope, or rate of change in VO_2_ per rate of change in *V*_*E*_, in 100 healthy volunteers during a ramped treadmill protocol from data truncated to RER levels from 0.85 to 1.2. The slope increased significantly from low-to-moderate intensity exercise and was highest at a RER of 1, decreasing at higher values. The O_2_ efficiency slope has been previously reported to be a valid and reproducible marker of function and prognosis as VO_2peak_. The present results show that VO_2_ slope is not constant during an incremental CPET and probably optimally measured at the RER of 1. How this tighter definition of the VO_2_ slope compares with VO_2_AT, *V*_*E*_/VCO_2_, ΔVO_2_/*W*, and chronotropic incompetence in the evaluation of patients with cardiac or pulmonary diseases will have to be investigated in further studies.

In summary, for those of us interested in a better understanding of the dyspnoea-fatigue symptoms of our patients, CPET is a remarkable tool. It generates lots of variables that need integration into pathophysiological reasoning and pretest clinical probability assessments. More work is needed for the integration of the CPET variables into validated decision trees and recommendations. This is an exciting area, still open for a lot more investigation and progress. The series papers published in this issue of Pulmonary Medicine are altogether an important step in the good direction.

*Luke Howard* *Luke Howard* *Michael P. W. Grocott* *Michael P. W. Grocott* *Robert Naeije* *Robert Naeije* *Ron Oudiz* *Ron Oudiz* *Roland Wensel* *Roland Wensel*

